# Anxiety makes time pass quicker while fear has no effect^☆^

**DOI:** 10.1016/j.cognition.2019.104116

**Published:** 2020-04

**Authors:** Ioannis Sarigiannidis, Christian Grillon, Monique Ernst, Jonathan P. Roiser, Oliver J. Robinson

**Affiliations:** a Institute of Cognitive Neuroscience, 17 Queen Square, University College London, London, WC1N 3AR, UK; b Section on Neurobiology of Fear and Anxiety, National Institutes of Health, 15K North Drive, Bethesda, MD 20814, United States

**Keywords:** Time perception, Anxiety, Threat-of-shock, Fear, Emotion

## Abstract

People often say that during unpleasant events, e.g. traumatic incidents such as car accidents, time slows down (i.e. time is overestimated). However aversive events can elicit at least two dissociable subtypes of reactions: fear (transient and relating to an imminent event) and anxiety (diffuse and relating to an unpredictable event). We hypothesised that anxiety might have an opposite effect on time perception compared to fear. To test this we combined a robust anxiety manipulation (threat-of-shock) with a widely used timing task in which participants judged whether the duration of a stimulus was long or short. In line with our hypothesis, across three experiments (with varying stimulus timings and shock levels), participants significantly underestimated time under inducted anxiety, as indicated by a rightward shift of the psychophysical function (meta-analytic effect size: d = 0.68, 95% confidence interval: 0.42-0.94). In two further studies, we were unable to replicate previous findings that fear leads to time overestimation, after adapting our temporal cognition task, which suggests a dissociation between fear and anxiety on how they affect time perception. Our results suggest that experimentally inducing anxiety leads to underestimating the duration of temporal intervals, which might be a starting point in explaining different subjective experiences of disorders related to fear (e.g. post-traumatic stress disorder) and anxiety (e.g. generalised anxiety disorder).

## 1 Introduction

The emotional valence of experience is thought to distort our subjective sense of time (Droit-Volet & Meck, 2007). Everyday experience and experimental evidence suggests that “time flies when having fun” (Gable & Poole, 2012; Simen & Matell, 2016), but slows down during unpleasant experiences (Fayolle, Gil, & Droit-Volet, 2015; Stetson, Fiesta, & Eagleman, 2007; Tipples, 2008). The latter seems to occur in situations such as car accidents or free falls (Arstila, 2012; Stetson et al., 2007). However, it might not be the case that time slows down during all kinds of aversive events. For example, time also seems to fly when one is in the rather anxiogenic state of having to work to a deadline just hours away.

One explanation for this discrepancy is that in the above examples during which time slows down (car accidents and well-controlled free falls: (Arstila, 2012; Stetson et al., 2007), it is *fear* that is induced (an acute aversive state elicited by immediate and certain threat), which is distinct from *anxiety* (a more prolonged aversive state elicited by an uncertain threat that may occur in the future; for further discussion of the distinction between fear anxiety see Davis, Walker, Miles, & Grillon, 2010; Kierkegaard, 1957; Tovote, Fadok, & Lüthi, 2015. The hypothesis that the passage of time slows down during states of fear (i.e. time is overestimated) is supported by studies using fear-provoking pictures (Grommet et al., 2011; Tipples, 2008, 2011), looming stimuli, which are considered intrinsic threat cues, (van Wassenhove, Wittmann, Craig, Bud, & Paulus, 2011), unpleasant noises (Droit-Volet, Mermillod, Cocenas-Silva, & Gil, 2010) and electrical shocks (Fayolle et al., 2015). An unresolved question, however, is whether the effect of anxiety on our perception of time is distinct from that of fear. One study that used electrical shocks hinted that this might be the case, though given that a probabilistic fear conditioning paradigm was used, it is not clear whether fear or anxiety was induced in this experiment (Lake, Meck, & LaBar, 2016).

During *fear*-inducing events (e.g. a car crash) attention is focused on timing the present. This could be adaptive: for example, when a car is speeding towards you, keeping track of time is critical, as taking evasive action at the right moment may allow you to avoid the collision. This is supported by experimental evidence which found that looming (inherently fear-inducing), but not receding stimuli, result in time overestimation (van Wassenhove et al., 2011). This increased attention to timing is thought to lead to overestimation (Thomas & Weaver, 1975), and is supported by experiments showing that individuals with greater susceptibility to fear (and thus increased attention to fear-related cues) overestimate the duration of fearful stimuli (Bar-Haim, Kerem, Lamy, & Zakay, 2010; Tipples, 2008). During *anxiety*, by contrast, attention is divided between what is happening at this moment and anticipating an uncertain aversive event that may happen in the near future. For example, imagine being an undergraduate student who has just started work on an assignment, the deadline for which is just three hours away: time seems to fly as the student is uncertain whether they will make the deadline. Whilst worrying about potentially missing the deadline, one is distracted from what is happening in the present moment (e.g. writing). It is thought that this type of distraction from time could lead to “missing ticks from our mental clock” (Coull, Vidal, Nazarian, & Macar, 2004; Macar, Grondin, & Casini, 1994; Thomas & Weaver, 1975). This explanation leads to the hypothesis, as yet untested, that anxiety – as distinct from fear – should result in *underestimating* time.

**Fig. 1:**
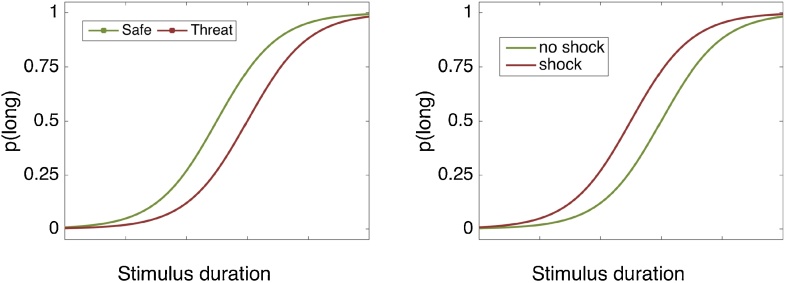
Predicted effect of anxiety (threat-of-shock) and fear on the temporal bisection task. The curves represent the proportion of long responses [p(long)] as a function of stimulus duration. (left) We predicted that the anxiety condition (threat) would promote a rightward shift of the curve, compared to the baseline (safe) condition, due to underestimation of time intervals. (right). In the fear condition, we predict a leftward shift of the curve compare to the baseline condition, due to overestimation of time intervals, replicating previous findings (Fayolle et al., 2015).

To test this hypothesis, we combined a commonly used timing task (Kopec & Brody, 2010) with an established anxiety manipulation: threat of shock (Robinson, Vytal, Cornwell, & Grillon, 2013; Schmitz & Grillon, 2012). During threat of shock, participants anticipate intermittent and unpredictable painful electrical stimulation to the skin over a prolonged period of time. This procedure reliably increases self-report, physiological and neurobiological indices of anxiety (Robinson et al., 2013; Schmitz & Grillon, 2012). Given the uncertain nature of the threat (which should elicit anxiety rather than fear), we hypothesised that participants would allocate attentional resources away from the timing task at hand and towards anticipating the next shock, which should lead to underestimation of time intervals (see Fig. 1).

This hypothesis was tested over the course of five separate experiments. In Experiment 1, participants performed a subsecond temporal bisection task under threat of shock and safe conditions. We predicted time underestimation due to increased anxiety, which should be evident in a rightward shift to this psychophysical curve (see Fig. 1). Since different mechanisms are considered to be involved in the estimation of subsecond compared to suprasecond durations (Buhusi & Meck, 2005; Koch et al., 2008), Experiment 2 sought to generalise these findings using suprasecond stimuli durations. Finally, given that in our threat of shock manipulation participants actually receive shocks, it is possible that any effects observed are due to the shocks *per se*, rather than the induced anxiety. Therefore Experiment 3 examined time estimation under threat, similar to Experiment 2, but without any shocks being delivered. In Experiment 4 & 5 we sought to replicate a previous study (Fayolle et al., 2015) showing that fear leads to time overestimation, using the same temporal cognition task used in Experiments 1, 2 & 3.

## 2 Procedure

### 2.1 Overview

During a single testing session, following written informed consent, participants initially completed questionnaires assessing their mood and anxiety levels, followed by a shock work-up procedure to determine an appropriate level of aversive electrical stimulation. Participants in Studies 1, 2 & 3 completed the temporal bisection task under an anxiety manipulation (threat of shock), while participants in Studies 4 & 5 completed the temporal bisection task under a fear manipulation. Information relating to participant recruitment and inclusion/exclusion criteria is provided in each of the experiment-specific methods sections below.

### 2.2 Apparatus

All experiment material was presented on Windows computers using Cogent 2000 (www.vislab.ucl.ac.uk/cogent.php; Wellcome Trust Centre for Neuroimaging and Institute of Cognitive Neuroscience, UCL, London), running under Matlab.

### 2.3 Self-report mood and anxiety questionnaires

Participants completed self-report measures of depression (Beck Depression Inventory: BDI (Beck & Steer, 1987) and trait anxiety (State Trait Anxiety Inventory: STAI (Spielberger, 1983)).

### 2.4 Shock calibration

A shock work-up procedure then followed in order to control for shock tolerance and skin resistance. Trains of shocks or single shocks (depending on the experiment) of different durations were delivered to the non-dominant wrist via a pair of silver chloride electrodes using a DS5 stimulator (Digitimer Ltd, Welwyn Garden City, UK). Participants received shocks sequentially with step increases in amplitude (starting with a low intensity and moving up), which they had to rate using a scale from 1 to 10 (1 meaning “I barely felt it” and 10 “shock is approaching the maximum level I can tolerate”). As soon as participants rated a shock as 10, the procedure was started over with using the intensity participants rated as 3. After this, the procedure was repeated one more time (three in total) starting with the intensity participants rated as 3 in the previous run. For each participant we used the intensity they rated as 8 throughout in this last run.

### 2.5 Stimuli

Our to-be-timed stimuli were emotional faces in order to replicate previous findings (Droit‐Volet, Brunot, & Niedenthal, 2004; Tipples, 2008, 2011) suggesting that emotional faces alter time perception. At the same time, we wanted to provide participants with interesting stimuli to time, in order to keep them engaged in the task and emotional faces are known to be preferentially accessed.

### 2.6 Temporal bisection task under threat of shock

**Fig. 2:**
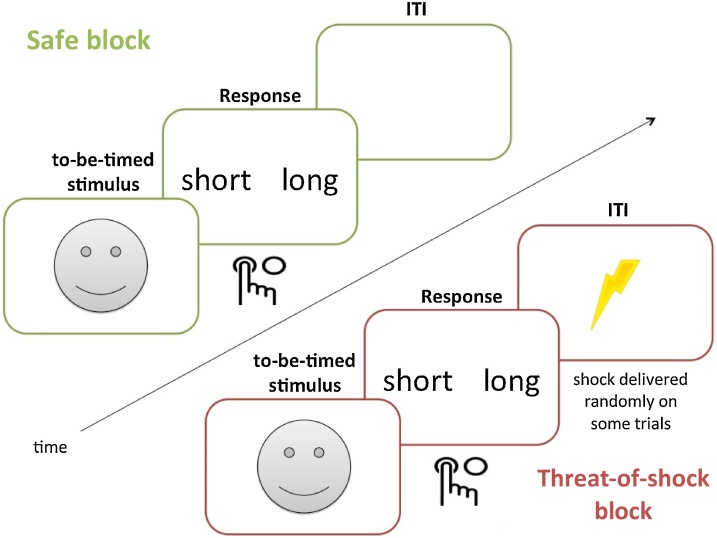
Task design for Experiments 1, 2 & 3, in which participants made time judgements during safe and threat of shock blocks. Note: in the actual experiment participants were presented with images from the NimStim Face Stimulus Set (happy neutral and fearful); smiley faces are presented here due to copyright issues.

In Experiments 1, 2, & 3, participants completed the visual temporal bisection task under two alternating conditions (Fig. 2): “threat-of-shock” (labelled “threat”), during which they could receive shocks at any time and without warning, and “safe” during which they could not receive any shocks (the order was counterbalanced). The task was flanked by coloured borders that indicated the condition (safe or threat), taken from a pool of four colours (red, blue, green, magenta), which was counterbalanced across participants, as was the order of threat and safe blocks.

A short training phrase preceded the main task. It consisted of presenting participants with two anchor durations (Fig. 2), a “short” duration (Study 1: 300 ms; Studies 2 & 3: 1,400 ms) and a “long” duration (Study 1: 700 ms; Studies 2 & 3: 2,600 ms). Each was presented three times, and presentation order was pseudorandomised. In addition, before the beginning of each block (safe or shock) the anchor durations were repeated.

On each trial, the to-be-timed stimuli were emotional facial expressions (happy, fearful and neutral) whose durations varied according to a predetermined range (Study 1: 300–700 ms, Studies 2 & 3: 1,400–2,600 ms; see also Table 1). Stimulus durations were pseudorandomised, and presented equally often in each threat and safe block to avoid potential biases (Wearden & Ferrara, 1996). On each trial participants were required to make a choice: press “short” if the duration of the stimulus was judged to be similar to the “short” anchor, or press “long” if the duration of the stimulus was judged to be similar to the “long” anchor (left and right buttons for these options were counterbalanced across participants). After the 1.5 s response limit, there was a variable inter-trial interval (ITI: three possibilities different for each experiment (see below), pseudo-randomised). Following each block, participants rated their anxiety levels using a continuous visual analogue scale.

**Table 1:** Experimental parameters.

	Stimulus duration	Manipulation	Shock occurrence	Shock type	Task duration
Study 1	300–700 ms	threat-of-shock (unpredictable)	immediately after response	train (3 s)	40 min
Study 2	1400–2,600 ms	threat-of-shock (unpredictable)	during ITI	train (2 s)	20 min
Study 3	1,400–2,600 ms	threat-of-shock (unpredictable)	no shock delivered	no shock	10 min
Study 4	1,400–2,600 ms	fear (predictable)	after stimulus disappeared	single pulse	10 min
Study 5	1,400–2,600 ms	fear (predictable)	after stimulus disappeared	train (0.5 s)	20 min

### 2.7 Temporal bisection task under fear

**Fig. 3:**
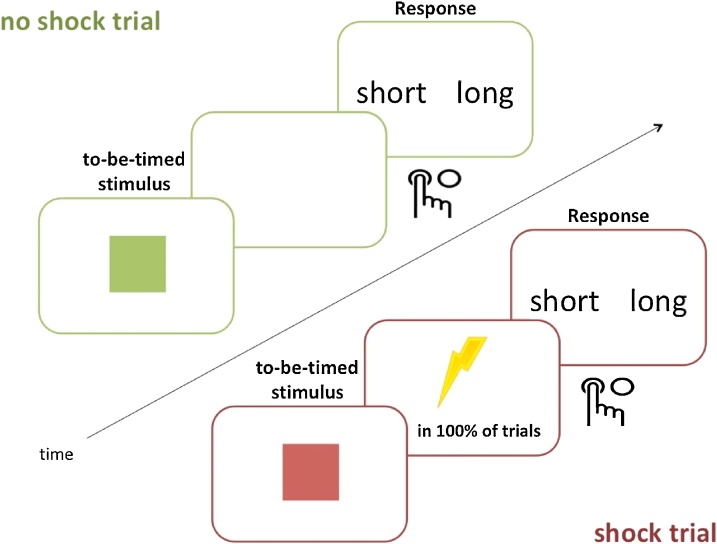
Task design for Experiments 4 & 5, in which participants made time judgements while alternating between shock and no shock trials. In the actual experiment participants were presented with blue and green fractal images indicating 100 % chance of shock or no shock (For interpretation of the references to colour in this figure legend, the reader is referred to the web version of this article).

In Experiments 4 & 5 participants completed a visual temporal bisection task in which they had to make judgements about the duration of pictures, similar to Experiments 1,2 & 3. In this task the colour of to-be-timed stimuli indicated whether participants would receive a shock (“shock” trials) or not (“no shock” trials). During “shock” trials participants always received a shock and during “no shock” trials they never received a shock. The shock was delivered as soon as the stimuli disappeared (Fig. 3). The order of the “shock” and “no-shock” trials was counterbalanced on each block.

A short training phrase preceded the main task. It consisted of presenting participants with two anchor durations a “short” duration (1,400 ms) and a “long” duration (2,600 ms). Each was presented three times, and presentation order was pseudorandomised. In addition, before the beginning of each block the anchor durations were repeated.

On each trial, the to-be-timed stimuli were fractals (blue or green) indicating “shock” or “no-shock” trials, whose durations varied according to a predetermined range (1,400–2,600 ms; see also Table 1) and counterbalanced across participants. Stimulus durations were pseudorandomised, and presented equally often in each threat and safe block to avoid potential biases (Wearden & Ferrara, 1996). On each trial participants were required to make a choice: press “short” if the duration of the stimulus was judged to be similar to the “short” anchor, or press “long” if the duration of the stimulus was judged to be similar to the “long” anchor (left and right buttons for these options were counterbalanced across participants). After the 1.5 s response limit, there was a variable inter-trial interval (2 s, 2.5 s and 3 s; pseudo-randomised).

### 2.8 Data analysis

All data was preprocessed in Matlab (v. R2015b), and statistical testing was carried out in SPSS (v. 23). The meta-analysis of the three studies was carried out in JASP (Version 0.8.6).

#### 2.8.1 Proportion of long responses

Trials on which participants did not make a response were excluded from the analysis. Repeated-measures analyses of variance (ANOVAs) were performed on the proportion of stimuli participants judged to be long (proportion of long responses, p(long). The effects of threat (safe or threat of shock condition), duration (six stimulus durations), the emotion depicted on the stimulus (fearful, happy or neutral) and block (where relevant) were used as within-subject factors. Experiment 3 consisted of only one safe and one threat block, and hence the ANOVA did not include block as a factor. Greenhouse-Geisser corrections were applied when violations of sphericity occurred.

#### 2.8.2 Psychophysical modelling

**Fig. 4:**
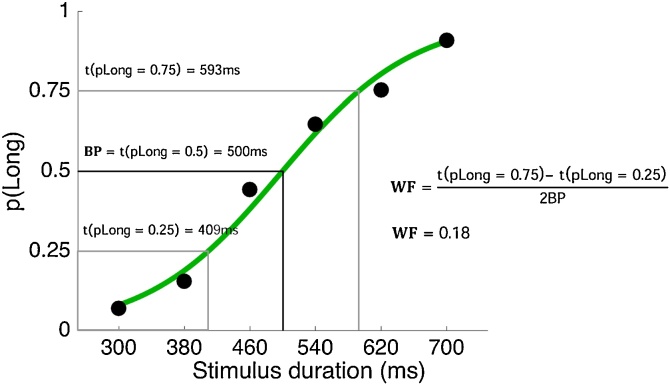
Example illustrating the calculation of the bisection point (BP) and Weber fraction (WF) (data from an exemplar participant in the safe condition in Study 1). p(Long)=proportion of stimuli classified as long; ms = milliseconds; t = stimulus duration that corresponds to p(Long) on the psychometric curve.

Next, for each participant we fitted psychometric functions to trials separately for the threat and safe conditions, and computed the bisection point (BP) and Weber fraction (WF) (Fig. 4). The BP is the time interval that is perceived to be equidistant between the shortest and longest anchor; i.e. the time interval corresponding to 50% pLong29. It provides a measure of the perceived duration of comparison intervals. A rightward shift of the psychophysical curve would lead to a greater BP, indicating underestimation of time (and vice-versa for a leftward shift). The WF is a measure of the precision of sensory discrimination (Kingdom & Prins, 2010). The more sensitive participants are to the task durations, the more quickly the curve will rise at its steepest point. A small WF indicates that small differences between the stimuli are detectable, in other words that sensitivity is higher. Paired samples t-tests were employed to compare BP and WF across the safe and threat conditions.

The data was modelled using the Palamedes toolbox in MATLAB (Prins & Kingdom, 2009). The proportion of long responses, pLong, at each comparison interval, was fitted with logistic functions defined by four parameters: threshold α, slope β, guess rate γ, and lapse rate λ. In line with previous studies, γ was fixed at 0 since the task was 2-alternative forced-choice; λ was fixed at 0.1 to allow for occasional attentional lapses (Terhune, Sullivan, & Simola, 2016). α and β were free parameters and estimated using maximum likelihood estimation. The duration corresponding to the 50 % threshold on the psychometric function was defined as the BP. To calculate the WF we calculated the difference between the durations corresponding to the 75% and 25% thresholds, and divided by twice the BP, or (t(pLong = 0.75) – t(pLong = 0.25))/2*BP), where t is the interval duration (x-axis in Fig. 4) at the respective location on the fitted psychometric function.

## 3 Experiment specific methods

### 3.1 Experiment 1: Threat of shock, subsecond durations

The study and all procedures were approved by the UCL Research Ethics Committee (Project ID Number: 1764/001) and were in accordance with the latest version of the Declaration of Helsinki. Participants were recruited from UCL subject databases (Table 2).

**Table 2:** Sample demographic information for the five studies. Figures represent counts or means (SDs).

	Sample size	Age	Female	BDI	STAI
Study 1	25	22.76 (0.67)	16	7.04 (1.92)	40.16 (2.66)
Study 2	25	23.36 (0.83)	13	4.96 (0.96)	36.24 (1.93)
Study 3	20	27.90 (6.42)	14	2.85 (2.30)	32.05 (6.51)
Study 4	25	24.28 (2.05)	18	6.16 (6.51)	40.08 (10.62)
Study 5	35	22.42 (2.83)	24	8.91 (8.62)	43.08 (11.91)

BDI = Beck depression inventory. STAI = Trait anxiety from the State Trait Anxiety Inventory.

A power calculation (G*power version 3.1.9.2 (Faul, Erdfelder, Lang, & Buchner, 2007)) determined the sample size based on the only study that fulfilled the following criteria: a) usage of a temporal bisection procedure as a timing task; b) manipulation of temporal cognition by delivering electric shocks (Fayolle et al., 2015). Only the experiment in which the to-be-judged durations were subsecond (from 0.2 to 0.8 s) was considered for the power calculation, since it was the closest to the stimulus duration range we used (0.3 to 0.7 s). This choice was made after considering that task properties might differ in sub- and supra-second temporal tasks Buhusi & Meck, 2005; Koch et al., 2008. The effect size (Cohen’s d) of the (Fayolle et al., 2015) study was calculated to be d = 1.89. This extremely large effect size may have occurred because on half of the trials participants always received a shock *while* timing a stimulus, and thus pattern of results might be due to the delivery of shocks *per se*. By contrast, in our threat of shock manipulation, shocks were delivered rather infrequently and occurred during ITIs. Hence, we conservatively decreased the Fayolle et al. (2015) effect size by 70 % to d = 0.56; with 80 % power and an alpha of 0.05 (two-tailed), the required sample size was estimated to be 25 participants.

Participants had normal or corrected to normal vision and no present (or past) neurological or psychiatric diagnosis. All provided written informed consent and received £10 for their participation, which lasted approximately 1 h and 20 min. Three participants’ data were not analysed due to incomplete data acquisition, and thus three extra participants had to be recruited to achieve a final sample of 25.

The session consisted of 18 blocks (nine in the safe and nine in the threat condition), with each block comprising 48 trials. On each trial, participants viewed a picture of an emotional face (happy, fearful or neutral; taken from the standardised NimStim (Tottenham et al., 2009) set) that remained on screen for 300, 380, 460, 540, 620 or 700 ms.

Seventy-two pictures were used in this experiment, depicting happy, neutral and fearful facial expressions, taken from 24 actors. During each block, participants viewed an equal number of happy, fearful and neutral facial expressions (16 each), the order of which was pseudorandomised. Similarly, stimulus durations were pseudorandomised within each block, so that all durations were repeated eight times. The ITI (500, 750, 1000 ms) was also pseudorandomised.

Participants received a total of 18 trains of shocks during the session, only during threat blocks, in the following combinations: four shocks in two blocks; three shocks in one block; two shocks in three blocks; one shock in one block. No shocks were delivered in two of the threat blocks. The order of the shocks was random for each participant and occurred on different trials, immediately following the participant’s response. Each train of shock consisted of 20 pulses delivered over 2 s and the average shock intensity was 5.99 mA (SD = 3.22). After each safe and threat block, participants had to rate how anxious they felt using a continuous visual analogue scale ranging from “very little” to “very much”.

### 3.2 Experiment 2: Threat of shock, suprasecond durations

The study and all procedures were approved by the UCL Research Ethics Committee (Project ID Number: 1764/001) and were in accordance with the latest version of the Declaration of Helsinki.

Participants were recruited from UCL subject databases. All had normal or corrected to normal vision, had no present or past neurological or psychiatric diagnosis. All provided written informed consent and received £10 for their participation, which lasted approximately 1 h. The same power calculation was used as for Experiment 1, requiring N = 25.

Each session consisted of 8 blocks, 4 safe and 4 threat, with each block comprising 48 trials. Similar to Experiment 1, on each trial participants viewed a picture of an emotional face (happy, fearful or neutral; taken from NimStim33), but which remained on screen for 1,400, 1,640, 1,880, 2,120, 2,360 or 2,600 ms. All other task aspects were identical to Experiment 1 apart from the number and duration of shocks as explained below.

Participants received between 5 and 11 shocks in total during the session, only during threat blocks. The shocks were randomly chosen from the following combinations: four shocks in one block, three shocks in one block, two shocks in two blocks, one shock in one block. No shocks were delivered in one of the threat blocks. The order of the shocks was random for each participant and occurred on different trials, at any time during the ITI. Each train of shock consisted of 30 pulses delivered over 3 s and the average shock strength was 10.01 mA (SD = 0.65). After each safe and threat block, participants had to rate how anxious they felt using a continuous visual analogue scale ranging from “very little” to “very much”.

### 3.3 Experiment 3: Threat without shocks, supra-second durations

The study and all procedures were approved by the NIH Institutional Review Board Project (ID Number: 01-M-0254) and were in accordance with the latest version of the Declaration of Helsinki.

Participants were recruited through advertisements (newspaper and public transport) in the Washington, D.C. metropolitan area. Following an initial telephone screen, participants visited the National Institutes of Health (NIH) for comprehensive screening by a clinician, which comprised a physical examination, urine drug screen, and the Structured Clinical Interview (SCID) for the Diagnostic and Statistical Manual of Mental Disorders (DSM), Fifth Edition (American Psychiatric Association, 2013). Exclusion criteria were: contraindicated medical disorder (i.e. those thought to interfere with brain function and/or behaviour); past or current psychiatric disorders; and use of psychoactive medications or recreational drugs (per urine screen). Twenty participants were tested (reduced from 25 in the first two experiments) which provided 80% power at an alpha of 0.05 (two-tailed) assuming the smallest effect of threat of shock detected in the first two experiments.

All participants provided written informed consent and were reimbursed $140 for their participation. Each session consisted of two blocks, one safe and one threat (counterbalanced) with each block comprising 48 trials. All other task parameters were identical to Experiment 2. Even though participants underwent the shock-work up (with shocks that lasted 200 ms), they did not receive any shocks during threat blocks. After each safe and threat block, participants had to rate how anxious they felt using a continuous visual analogue scale ranging from “very little” to “very much”.

### 3.4 Experiments 4 & 5: Fear manipulation, supra-second durations

The study and all procedures were approved by the UCL Research Ethics Committee (Project ID Number: 1227/001) and were in accordance with the latest version of the Declaration of Helsinki.

A power calculation (G*power version 3.1.9.2 (Faul et al., 2007)) determined the sample size of Study 4 based on the averaged effect size of Studies 1, 2, & 3. Thus, to achieve an effect size d = 0.68 with 90% power and an alpha of 0.05 (two-tailed), the required sample size was estimated to be 25 participants. Given that the results of Study 4 were trending towards significance, we run Study 5 in which the expected effect size was d = 0.50 and with 90% power and an alpha of 0.05 (two-tailed), the required sample size was estimated to be 35 participants.

Participants had normal or corrected to normal vision and no present (or past) neurological or psychiatric diagnosis. All provided written informed consent and received £4 in Study 4 and £6 in Study 5 for their participation.

The session consisted of 2 blocks in Study 4 and 4 blocks in Study 5 with each block comprising 48 trials. On each trial, participants viewed a fractal image that remained on screen for 1,400, 1,640, 1,880, 2,120, 2,360 or 2,600 ms.

Forty-eight pictures were used in this experiment, depicting twenty-four green and twenty-four blue fractal images, each presented once per block, with their order pseudorandomised. Similarly, stimulus durations were pseudorandomised within each block, so that all durations were repeated eight times. The ITI (2 s, 2.5 s and 3 s) was also pseudorandomised.

Participants received a total of forty-eight shocks during the session, only during shock trials. No shocks were delivered in the no-shock trials. In Experiment 4 the shock consisted of a single pulse and in Experiment 5 of 5 pulses. At the end of each experiment, participants were asked to verbally report how unpleasant and pleasant they found each the shocks using a scale from 1 to 10, where 1 means “very little” and 10 “very much”

## 4 Results

### 4.1 Study 1: Threat-of-shock, subsecond durations

#### 4.1.1 Manipulation check: subjective ratings

**Fig. 5:**
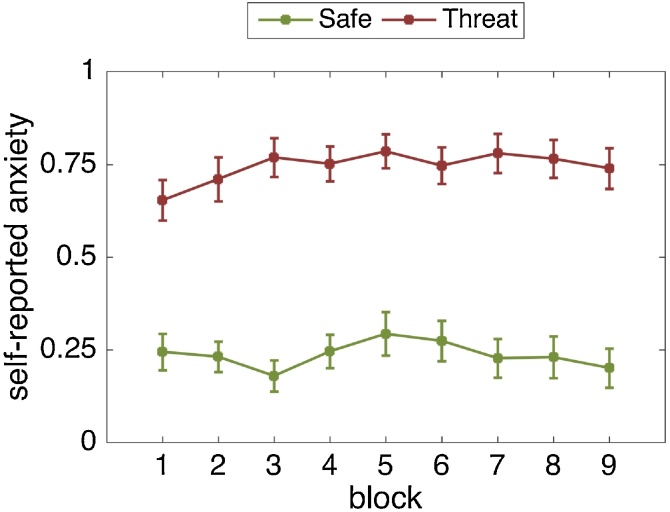
Self-reported anxiety levels on each block per threat condition. Greater values reflect higher anxiety. Error bars are standard errors of the mean (SEM).

Across blocks, participants reported being significantly more anxious in the threat (M = 0.74, SD = 0.22) compared to the safe (M = 0.24, SD = 0.20) condition (F(1, 24) = 77.10, p < .001, η_p_2 = .763). The effect of block (F(4.24, 101.77) = 1.59, p = .180, η_p_2 = .062) and the threat-by-block interaction were non-significant (F(8, 192) = 1.51 p = .156, η_p_2 = .059). Hence, self-reported anxiety was elevated during the threat condition, and this effect was stable throughout the experiment (Fig. 5).

#### 4.1.2 Time estimation: proportion of long responses

For this ANOVA analysis, data from five participants were excluded, as there were missing responses in some conditions due to the experiment being quite long and repetitive, but this is not an issue for the curve fitting (see Bisection Point analysis below), which should therefore be considered with more confidence.

**Fig. 6:**
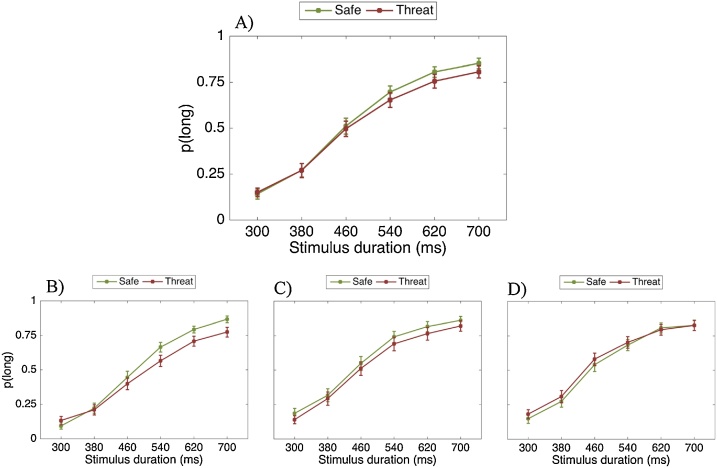
Proportion of stimuli rated “long” as a function of the actual presentation length and threat condition. A: All 18 blocks | B: First six blocks | C: Middle six blocks | D: Final six blocks. Error bars are standard errors of the mean (SEM).

There was a significant main effect of stimulus duration (F(2.08, 39.58) = 222.07, p < .001, η_p_2 = .914). As expected, the longer the stimulus duration, the more likely it was to be classified as “long” (Fig. 6). All interactions with stimulus duration were non-significant. There was a significant main effect of threat (F(1, 19) = 6.10, p = .023, η_p_2 = .243) but not of emotion (F(2, 38) = 0.61, p = .550, η_p_2 = .031), and the threat-by-emotion interaction was non-significant (F(2, 38) = 0.07, p = .936, η_p_2 = .003). Participants made significantly more “long” choices as the experiment progressed (main effect of block: F(8, 152) = 6.54, p < .001, η_p_2 = .256) and the threat-by-block interaction was also significant (F(8, 152) = 2.66, p = .009, η_p_2 = .123). This suggests that participants’ temporal perception and the effect of threat changed over the course of the experiment.

To make the above results easier to interpret, we performed a *post-hoc* analysis binning data into three experimental stages (first six blocks, middle six blocks, final six blocks). The effect of threat was significant for the first six blocks, (F(1, 24) = 7.10, p = .014, η_p_2 = .228) and, the middle six blocks (F(1, 24) = 12.69, p = .002, η_p_2 = .346), but not for the final six blocks (F(1, 24) = 1.81 p = .191, η_p_2 = .070). As shown in Fig. 6, participants underestimated time under threat-of-shock and this effect disappeared during the final blocks of the experiment.

#### 4.1.3 Psychophysical modelling

##### 4.1.3.1 Bisection point

Averaged across blocks, the BP was significantly larger during the threat (M = 483.83, SD = 116.20) compared to the safe (M = 456.86, SD = 107.20) condition (t(24) = 3.79, p = .001, d = 0.76). This indicates that in the threat condition the psychometric curve was shifted to the right and thus time was underestimated.

##### 4.1.3.2 Weber fraction

Averaged across blocks, WF was not significantly different between the threat (M = 0.25, SD = 0.02) and safe (M = 0.23, SD = 0.02) conditions (t(24) = 1.26, p = .221, d = 0.25). Thus there was no evidence that the sensitivity to time intervals differed between the safe and threat conditions.

### 4.2 Study 2: Threat-of-shock, suprasecond durations

#### 4.2.1 Manipulation check: anxiety ratings

**Fig. 7:**
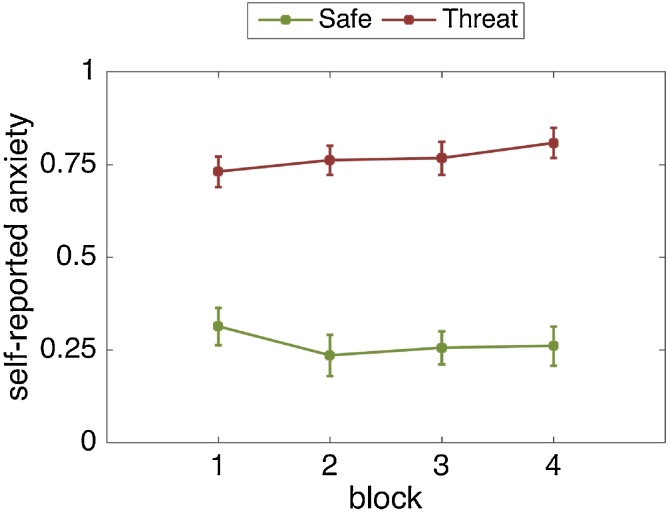
Self-reported anxiety levels on each block per threat condition. Greater values reflect higher anxiety. Error bars are standard errors of the mean (SEM).

Across blocks, participants reported being significantly more anxious in the threat (M = 0.76, SD = 0.17) compared to the safe (M = 0.26, SD = 0.22) condition (F(1, 24) = 97.81, p < .001, η_p_2 = .803). The effect of block was non-significant (F(1.99, 47.83) = .458, p = .634, η_p_2 = .019). but the threat-by-block interaction was significant (F(3, 72) = 3.17, p = .029, η_p_2 = .117). Self-reported anxiety was strongly elevated during the threat condition relative to the safe condition, and this effect increased over time (Fig. 7). This is because individuals became even less anxious in the safe condition while at the same time becoming slightly more anxious in the threat condition.

#### 4.2.2 Time estimation: proportion of long responses

**Fig. 8:**
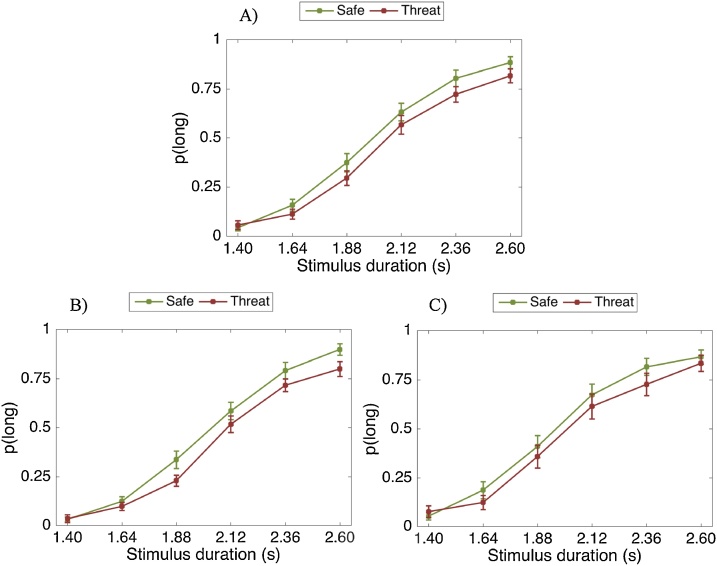
Proportion of stimuli rated “long” as a function of the actual presentation length and threat condition. A: All eight blocks | B: First four blocks | C: Final four blocks. Error bars are standard errors of the mean (SEM).

For this analysis, data from one participant was excluded, as there were missing responses in some conditions. There was a significant main effect of stimulus duration (F(2.06, 47.46) = 217.33, p < .001, η_p_2 = .904). As expected, the longer the stimulus duration, the more likely it was to be classified as “long” (Fig. 8). The threat-by-stimulus duration interaction was significant (F(3.23, 74.25) = 3.57, p = .016, η_p_2 = .134). As shown in Fig. 8, the threat manipulation mainly affected the longer stimulus durations. All other interactions with stimulus duration were non-significant.

There was a significant main effect of threat (F(1, 23) = 13.05, p = .001, η_p_2 = .362) but not of emotion (F(2, 46) = 2.09, p = .136, η_p_2 = .083). The threat-by-emotion interaction was non-significant (F(2, 46) = 0.02, p = .980, η_p_2 = .001). Participants made significantly more “long” choices as the experiment progressed (main effect of block: F(3, 69) = 4.70, p = .005, η_p_2 = .170) but the threat-by-block interaction was not significant (F(3, 69) = 1.34, p = .251, η_p_2 = .057). This suggests that participants’ temporal perception changed over time regardless of threat condition. This differs from Study 1, in which the effect of threat decreased over the course of the experiment.

#### 4.2.3 Psychophysics modelling

##### 4.2.3.1 Bisection point

Averaged across blocks, the BP was significantly larger during the threat (M = 2,123.74, SD = 271.66) compared to the safe (M = 2,035.62, SD = 276.29) condition (t(24) = 3.39, p = .002, d = 0.68). This indicates that under threat, the psychometric curve was shifted to the right and thus time was underestimated.

##### 4.2.3.2 Weber fraction

Averaged across blocks, the WF was not significantly different between the threat (M = 0.13, SD = 0.02) and safe (M = 0.11, SD = 0.01) conditions (t(24) = 1.77, p = .088, d = 0.35). Thus there was no evidence that the sensitivity to time intervals differed between the safe and threat conditions.

### 4.3 Study 3: Threat-without-shocks, supra-second durations

#### 4.3.1 Manipulation check: anxiety ratings

Despite the lack of shocks, across blocks, participants reported being more anxious in the threat (M = 0.36, SD = 0.27) compared to the safe (M = 0.11, SD = 0.17) condition (t(19) = 4.37, p < .001, d = 0.98).

#### 4.3.2 Proportion of long responses

**Fig. 9:**
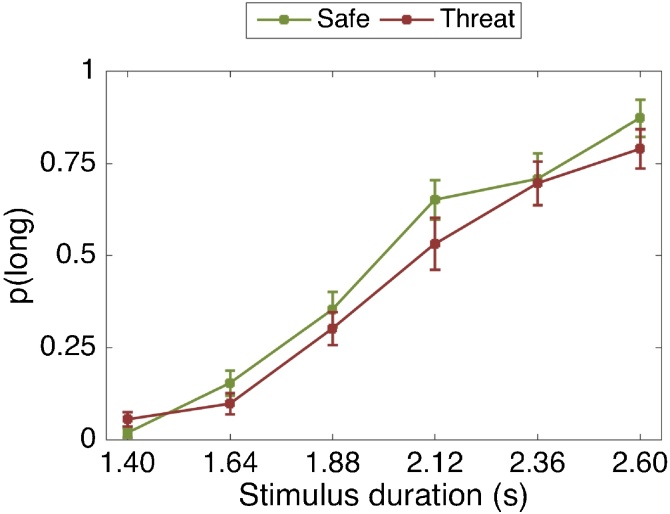
Proportion of stimuli rated “long” as a function of the actual presentation length and threat condition (one safe vs. one threat block). Error bars are standard errors of the mean (SEM).

There was a significant main effect of stimulus duration (F(2.56, 48.75) = 116.93 p < .001, η_p_2 = .860). As expected, the longer the stimulus duration, the more likely it was to be classified as “long” (Fig. 9). All interactions with stimulus duration were non-significant.

There was a significant main effect of threat (F(1, 19) = 6.88, p = .017, η_p_2 = .266), but not of emotion (F(2, 38) = 0.73, p = .487, η_p_2 = .037), and the threat-by-emotion interaction was non-significant (F(2, 38)<0.01, p = .997, η_p_2<.001). These results, which are consistent with the first two studies, suggest that time was underestimated in a threatening situation (Fig. 9), even though participants did not actually receive any shocks.

#### 4.3.3 Psychophysical modelling

##### 4.3.3.1 Bisection point analysis

The BP was significantly larger during the threat (M = 2,121.34, SD = 246.75) compared to the safe (M = 2,027.04, SD = 186.19) condition (t(19) = 2.66, p = .015, d = 0.60). This indicates that under threat, the psychometric curve was shifted to the right and thus time was underestimated.

##### 4.3.3.2 Weber fraction analysis

The WF was not significantly different between the threat (M = 0.31, SD = 0.21) and safe (M = 0.29, SD = 0.13) conditions (t(19) = 0.48, p = .639, d = 0.11). Thus there was no evidence that the sensitivity to time intervals differed between the safe and threat conditions.

### 4.4 Exploratory combined on the effect of emotion

In contrast to previous studies (Droit‐Volet et al., 2004; Tipples, 2008, 2011), we did not detect a significant effect of the facial expression of the to-be-timed stimulus (fearful, happy or neutral) on participants’ temporal judgements. One possibility is that our studies were underpowered to detect such differences, given that previous studies used larger sample sizes (the original study by Droit-Volet et al. 2004 used 37 participants, while an indirect replication by Tipples, 2008 used 43). Hence we pooled data together from Studies 1, 2 and 3 (combined N = 70) and performed a repeated-measures ANOVA on p(long) with threat, emotion and stimuli duration as within subject factors. This analysis revealed a significant effect of threat (F(1, 69) = 28.59, p < .001, η_p_2 = .293) but not of emotion (F(2, 138) = 1.59, p = .208, η_p_2 = .023). Thus, taken together, our studies suggest that the emotion depicted on the to-be-timed-stimuli did not affect the perception of time.

### 4.5 Meta-analysis of the effect of anxiety on time perception

**Fig. 10:**
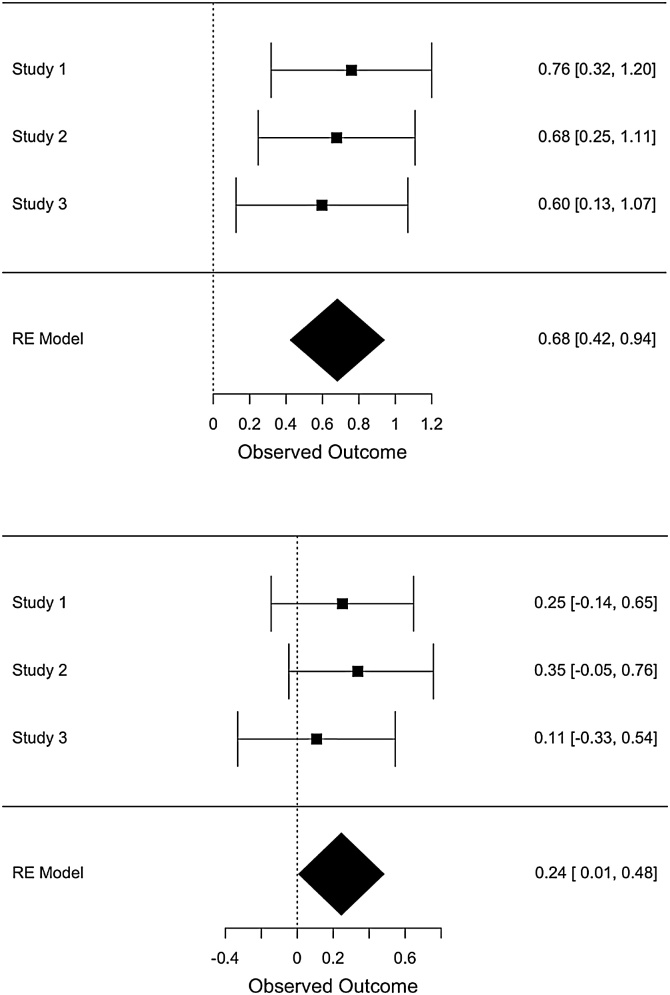
Meta-analytic results for the difference in bisection point (top panel) and Weber’s Fraction (bottom panel) between threat and safe conditions. The measure of effect size is Cohen’s d (standardised mean difference). RE = random effects.

A meta-analysis was conducted across the three experiments to quantify more precisely the effect of threat on temporal perception (Fig. 10). The difference in BP and WF between the threat and safe conditions was used as the metric to represent the effect of the anxiety manipulation on time perception.

There was a significant effect of threat on the BP (Z = 5.18, p < .001) and WF (Z = 2.02, p = 0.043) The meta-analytic effect size for the BP was d = 0.67 (95% confidence interval: 0.42-0.94) and for WF d = 0.24 (95% confidence interval: 0.01-0.48).

### 4.6 Study 4: Fear, suprasecond durations

#### 4.6.1 Manipulation check: shock ratings

After the end of the experiment participants were asked to rate on a scale 1–10 how pleasant and unpleasant they found the shocks, where 1 corresponds to very little and 10 very much. Overall, participants rated the shocks as more unpleasant (M = 6.52, SD = 1.92) than pleasant (M = 2.64, SD = 1.62) and the difference was statistically significant (t(24) = 6.99, p < .001, d = 1.4).

#### 4.6.2 Time estimation: proportion of long responses

**Fig. 11:**
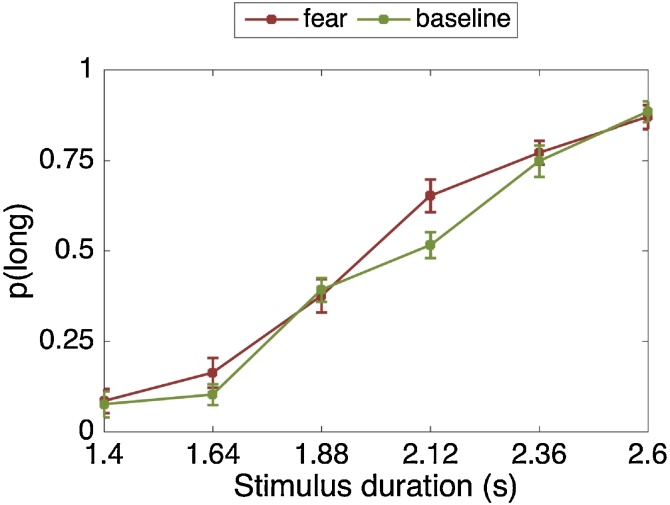
Proportion of stimuli rated “long” as a function of the actual presentation length and fear condition. Error bars are standard errors of the mean (SEM).

There was a significant main effect of stimulus duration (F(2.13, 51.26) = 124.63, p < .001, η_p_2 = .839). As expected, the longer the stimulus duration, the more likely it was to be classified as “long” (Fig. 11). The fear-by-stimulus duration interaction was not significant (F(5, 120) = 1.98, p = .086, η_p_2 = .076). As shown in Fig. 11, the fear manipulation mainly affected the longer stimulus durations. All other interactions with stimulus duration were non-significant.

The main effect of fear (F(1, 24) = 3.78, p = .063, η_p_2 = .136) was not significant, but was approaching significance. Thus, it is not clear whether participants’ temporal perception changed due to the fear manipulation. Regardless, the direction of the results are opposite to that of Studies 1, 2 & 3 in which during the anxiety manipulation time was underestimated. Our results show that, unlike anxiety, the fear manipulation does not make participants underestimate the temporal intervals.

#### 4.6.3 Psychophysics modelling

##### 4.6.3.1 Bisection point

Averaged across blocks, the BP was smaller during the fear (M = 2,004.77, SD = 194.98) compared to the baseline (M = 2,083.58, SD = 153.06) condition but the effect was again non-significant (t(24) = 1.99, p = .058, d = 0.40). There is nevertheless a trend that under fear, the psychometric curve was shifted to the left and thus time was overestimated, while the curve was shifted to the right in Studies 1, 2 & 3.

##### 4.6.3.2 Weber fraction

Averaged across blocks, the WF was not significantly different between the fear (M = 0.16, SD = 0.16) and baseline (M = 0.15, SD = 0.11) conditions (t(24)=−.38, p = .707, d = 0.01). Thus there was no evidence that the sensitivity to time intervals differed between the baseline and fear conditions.

### 4.7 Study 5: Fear, suprasecond durations

#### 4.7.1 Manipulation check: shock ratings

After the end of the experiment participants were asked to rate on a scale 1–10 how pleasant and unpleasant they found the shocks, where 1 corresponds to very little and 10 very much. Overall, participants rated the shocks as more unpleasant (M = 6.72, SD = 1.33) than pleasant (M = 2.96, SD = 1.81) and the difference was statistically significant (t(34) = 9.13, p < .001, d = 1.54).

#### 4.7.2 Time estimation: proportion of long responses

**Fig. 12:**
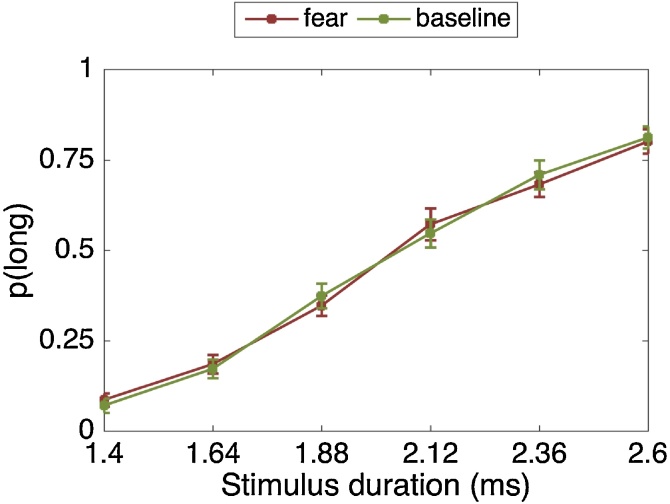
Proportion of stimuli rated “long” as a function of the actual presentation length and fear condition. Error bars are standard errors of the mean (SEM).

There was a significant main effect of stimulus duration (F(2.49, 84.87) = 142.77, p < .001, η_p_2 = .808). As expected, the longer the stimulus duration, the more likely it was to be classified as “long” (Fig. 12). The fear-by-stimulus duration interaction not significant (F(5, 170) = 0.57, p = .720, η_p_2 = .017).

The main effect of fear (F(1, 34) = 0.02, p = .888, η_p_2 = .001) was not significant.

#### 4.7.3 Psychophysics modelling

##### 4.7.3.1 Bisection point

Averaged across blocks, the BP during the fear (M = 2,129.33, SD = 316.05) compared to the baseline (M = 2,150.82, SD = 331.25) condition did not differ statistically (t(34) = 0.41, p = .687, d = 0.06).

##### 4.7.3.2 Weber fraction

Averaged across blocks, the WF was not significantly different between the fear (M = 0.18, SD = 0.10) and baseline (M = 0.17, SD = 0.13) conditions (t(34)=−.27, p = .791, d = 0.04). Thus there was no evidence that the sensitivity to time intervals differed between the baseline and fear conditions.

## 5 Discussion

Our results suggest that experimentally inducing anxiety leads to underestimating the duration of temporal intervals. Hence, although prior studies indicated that time slows down during aversive events (Arstila, 2012; Fayolle et al., 2015; Stetson et al., 2007; Tipples, 2008), this might be true only for events in which there is imminent threat, which induce a fearful (rather than anxious) state (Davis et al., 2010). At the same time, we failed to replicate the findings of a previous study that provided clear evidence that fear leads to overestimation of temporal intervals, suggesting a dissociation on how fear and anxiety affect time perception.

### 5.1 Effect of anxiety on time perception

In Studies 1–3, we found that participants underestimated time when anxious (i.e. their psychometric curve shifted to the right), while we did not detect a significant effect on temporal sensitivity (WF did not differ statistically between the threat and safe conditions). This indicates that under our anxiety manipulation, the perception of time was biased, but the sensitivity to the time intervals was not affected *per se*. In other words, anxiety did not impair the ability to discriminate between different time intervals, but only led participants to *perceive* the time intervals as shorter. In the meta-analysis we conducted, pooling together effects from Studies 1–3, we found both a significant effect of anxiety both in BP and WF. The latter effect was however relatively small (d = 0.24) and barely significant (Z = 2.02, p = 0.043), thus we cannot conclude with confidence that anxiety shifted WF.

A limitation in Studies 1 & 2 – that participants received shocks in the anxiety condition compared to the baseline – was addressed by Study 3. The latter showed that participants still underestimated time even though they did not receive shocks during the anxiety manipulation. This suggests that the effect of threat on time perception cannot be attributed solely to the physical properties of the shocks that participants received. In fact, previous studies showed that pain results in the opposite effect to what we found i.e. temporal overestimation (Rey et al., 2017). Thus, being in the anxiogenic state of anticipating an unpleasant event (without it happening), seems sufficient to bias the perception of time.

Regarding Studies 1 & 2, although intuitively it may seem that sub and supra-second durations used in these experiments respectively are almost identical in the neurocognitive level, there is considerable work suggesting that different mechanisms are responsible for both (Buhusi & Meck, 2005; Koch et al., 2008; Wiener, Turkeltaub, & Coslett, 2010), and so replicating the effect across both time is an important demonstration of generalizability.

Our meta-analytic effect size is d = 0.68, and although large for psychology standards, in real-life situations it might actually be even larger. During prolonged periods of anxiety e.g. during an exam, someone might feel like they still have plenty of time left, while in reality it is time to turn in their paper.

### 5.2 Effect of fear on time perception

Taking together the findings from Studies 4 & 5, the evidence is not strong enough to argue that our fear manipulation shifted time perception and thus we are unable to reject the null hypothesis. Although in Study 4 participants seemed to be overestimating temporal durations (in line with our hypothesis) the effect was only trending significance. In the follow-up Study 5 in which power was increased (by a. increasing the number of participants b. increasing the number of trials c. and by making the manipulation stronger i.e. the shocks more aversive) there was no evidence of temporal overestimation. At the same time, in none of these studies did we detect an effect of fear on temporal sensitivity (WF did not differ statistically between the fear and baseline trials). These results are not in line with a previous study that used a similar design to ours (Fayolle et al., 2015) and found a rather large effect of fear on time perception (d = 1.89). A key difference is that in their study, the shock was received at any point during which the to-be-timed stimuli was on. While in our study, the shock was delivered only *after* the to-be-timed stimulus disappeared from the screen. Thus, in this other study (Fayolle et al., 2015), it is not clear whether it is fear or the recovery from an negative event that has just happened that led to overestimating time. In both of these studies, shocks were delivered on 100% of the shock trials, which leaves open the possibility that participants habituated to the shocks per se very quickly, and thus putting into question whether our fear manipulation was successful. Future studies could try replicating our results by decreasing the shock reinforcement to 60 %, in line with that is being used in the fear conditioning literature. Nevertheless, this null result provides a compelling indication that our anxiety findings are not a ‘generic’ effect of aversive stimuli and instead potentially specific to unpredictable threats (i.e. anxiety).

### 5.3 Hypothesised mechanism behind the effect of fear and anxiety on time perception

Our results provide a more refined understanding of how aversive mental states might influence how we perceive time, and are consistent with the notion that anxiety and fear have opposing effects. The way each of these mental states affect our perception of time can be explained considering attention-based models of time perception (Thomas & Weaver, 1975), as well as experimental work (Coull et al., 2004; Failing & Theeuwes, 2016; Macar et al., 1994; Thomas & Weaver, 1975; Tse et al., 2004; Wearden, O’Rourke, Matchwick, Min, & Maeers, 2010). Specifically, during a fearful event, e.g. a car accident, it is adaptive to focus attention on the threatening object, including its timing properties. In the example of a car crash, paying close attention to the timing properties of the car driving towards you makes it more likely that you will turn the right moment to avoid the collision. Thus, it is possible that increased attention paid to timing the threatening object during a fearful state leads to time overestimation. The same attentional mechanism might be in place in general for surprising or novel experiences. Experimental studies suggest that surprising events, which automatically capture attention (Horstmann, 2015), lead to a slower perception of the passage of time i.e. time overestimation. This has been well documented in oddball paradigms, in which repetitive “standard” stimuli are interrupted by a deviant “oddball” stimulus, with the latter automatically attracting attention. In such studies, the oddball stimuli were consistently judged as longer compared to the standard stimuli (Birngruber, Schröter, & Ulrich, 2014; Failing & Theeuwes, 2016; Tse, Intriligator, Rivest, & Cavanagh, 2004), consistent with the hypothesis that increased attention allocation to the surprising stimulus leads to time overestimation.

While during fearful events attention is focused on the situation occurring at the present moment, in anxiety attention may be shifted away from the task at hand, towards threatening events that could happen in the future (e.g. while trying to finish an essay, worry about potentially missing the looming deadline). In that sense, timing under anxiety could be compared to psychological studies during which participants perform two competing tasks simultaneously. Such tasks have showed that the subjective duration of stimuli is increasingly underestimated the more participants attend to nontemporal stimulus features, such as form, color (Coull et al., 2004; Hicks, Miller, Gaes, & Bierman, 1977) or semantic meaning (Macar et al., 1994). In anxiety, *decreased* attention is directed towards what is happening right now, including timing the present and towards anticipating a negative effect to occur. Given that decreased attention to time has been associated with a feeling that time flies i.e. time underestimation (Block, Hancock, & Zakay, 2010), this might explain our finding that anxiety leads to time underestimation.

Previous studies suggest an association between interval timing and arousal i.e. that stimuli eliciting an arousal response are typically perceived as being longer in duration (for a review see (Lake, LaBar, & Meck, 2016)). Given our paradigm, it is reasonable to conjecture that our effects are driven partly by arousal since anxiety leads to increased arousal. However, our principal result (i.e. time underestimation) is opposite to what is typically observed for arousing stimuli, and so we conclude that our results are most parsimoniously explained by an attentional mechanism.

Our results therefore might help explain different subjective experiences in fear and anxiety disorders. On the one hand, in specific phobias and post-traumatic stress disorder, it might be the fear component that leads to the feeling that time slows down (Vicario & Felmingham, 2018). On the other hand, in generalised anxiety, given that individuals anticipate that negative events could occur at any moment, and thus their attention is always distracted from what is happening right now, this might leave them with a sense that time flies (Mioni, Stablum, Prunetti, & Grondin, 2016).

### 5.4 Effect of emotional faces on time perception

In contrast with previous studies (Droit-Volet & Meck, 2007), we did not find that emotional pictures affected the perception of time. Pooling data from all our studies together to increase power also failed to show an effect on emotion, despite a clear impact of induced anxiety. One possible explanation for the surprising pattern of results is that anxiety (including the low levels of anxiety experienced during the safe condition) swamped any impact of the emotional faces. In other words, the emotional impact of viewing happy and fearful faces may have been minimised since participants were in an overall anxiogenic context, anticipating possible painful stimulation. Taking into account that anxiety has been shown to induce an egocentric mindset when inferring other’s mental state (Todd, Forstmann, Burgmer, Brooks, & Galinsky, 2015) a related possibility is that participants were less likely to be influenced by the facial expressions they observed, since they were anxious. A final possibility is that the effect of emotional faces on time perception is confounded by perceptual complexity (Folta-Schoofs, Wolf, Treue, & Schoofs, 2014; Palumbo, Ogden, Makin, & Bertamini, 2014) and novelty (Cai, Eagleman, & Ma, 2015) and thus might depend on the particular stimuli used. Further experiments investigating the effect of emotional pictures on the perception of time should control for these confounds.

### 5.5 Limitations

We note that for Study 2, we used the same exact power calculation as in Study 1. Our aim was to replicate the findings from Study 1 by just changing one parameter, the stimulus durations (suprasecond durations instead of subsecond). If we were to replicate our results, we would need 25 participants assuming our meta-analytic d = 0.68, with 90 % power and an alpha of 0.05 (two-tailed).

While we found evidence that anxiety leads to underestimating time, our results do not support that fear leads to time overestimation. However, the extant literature contains a number of studies suggesting that fear leads to time overestimation (Droit-Volet et al., 2010; Fayolle et al., 2015; Grommet et al., 2011; Tipples, 2011). Another limitation is that in all our studies we used emotional pictures, and thus it is not clear whether our effect generalises to timing non-emotional events. Even though we did not find an effect of emotion (happy, fearful and neutral expression) on time perception, a future study could attempt to replicate our results using neutral pictures (for example, fractal images) to confirm that this effect is not related to the timing of faces *per se*.

It is also worth noting that in this study, consistent with the translational animal literature (Davis et al., 2010), we frame anxiety as response to an unpredictable, but known, aversive outcome. Anxiety may, nevertheless, be exacerbated by unknown aversive outcomes, actual or imagined (e.g. catastrophizing); a hypothesis that may be tested in further research.

We should note that the present paper was designed to better understand the cognitive effects of anxiety (with an ultimate aim to help us better understand a prominent mental health condition) rather than better understand the mechanisms of time-perception. Overall our results are in line with an attention-based account of how anxiety affects time perception. Nevertheless, considerably more work is needed to help us better understand the fundamental mechanisms of time perception.

## 6 Conclusion

In contrast to previous studies suggesting that unpleasant events induce a state during which the passage of time slows down (Fayolle et al., 2015; Stetson et al., 2007; Tipples, 2008, (Bar-Haim et al., 2010; Grommet et al., 2011; Tipples, 2011), we found that anxiety is associated with temporal under-estimation, i.e. that time flies. Grounded in contemporary conceptualisations highlighting the dissociation between fear and anxiety, we suggest that in prior studies in which an aversive event led to temporal over-estimation, it was fear that was induced, rather than anxiety. We argue that this dissociation between the effects of fear and anxiety on time perception might be explained based on attentional accounts of time perception and that it might enable better understanding of the symptoms of fear- vs. anxiety- specific pathological states.

## Author contributions

I. Sarigiannidis developed the study concept. He designed the study with input from O. J. Robinson and J. P. Roiser. Testing and data collection were performed by I. Sarigiannidis under the supervision of O. J. Robinson and J. P. Roiser at UCL (Study 1, 2, 4, 5), and Christian Grillon and Monique Ernst at NIH (Study 3). Data analysis and interpretation was carried out by I. Sarigiannidis, O. J. Robinson and J. P. Roiser. I. Sarigiannidis drafted the manuscript with critical revisions from O. J. Robinson and J. P. Roiser. Christian Grillon and Monique Ernst provided useful comments to this draft. All authors approved the final version of the manuscript for submission. We would like to thank Dr Devin Terhune, Professor Vincent Walsh, and Dr William Skylark for useful discussions that helped shape this manuscript.

## Declaration of Competing Interest

None.

## References

[bib0005] American Psychiatric Association (2013). American Psychiatric Association & DSM-5 Task Force. Diagnostic and statistical manual of mental disorders: DSM-5.

[bib0010] Arstila V. (2012). Time slows down during accidents. Frontiers in Psychology.

[bib0015] Bar-Haim Y., Kerem A., Lamy D., Zakay D. (2010). When time slows down: The influence of threat on time perception in anxiety. Cognition & Emotion.

[bib0020] Beck A.T., Steer R.A. (1987). BDI, Beck depression inventory: Manual.

[bib0025] Birngruber T., Schröter H., Ulrich R. (2014). Duration perception of visual and auditory oddball stimuli: Does judgment task modulate the temporal oddball effect?. Attention, Perception & Psychophysics.

[bib0030] Block R.A., Hancock P.A., Zakay D. (2010). How cognitive load affects duration judgments: A meta-analytic review. Acta Psychology (Amst.).

[bib0035] Buhusi C.V., Meck W.H. (2005). What makes us tick? Functional and neural mechanisms of interval timing. Nature Reviews Neuroscience.

[bib0040] Cai M.B., Eagleman D.M., Ma W.J. (2015). Perceived duration is reduced by repetition but not by high-level expectation. Journal of Vision.

[bib0045] Coull J.T., Vidal F., Nazarian B., Macar F. (2004). Functional anatomy of the attentional modulation of time estimation. Science.

[bib0050] Davis M., Walker D.L., Miles L., Grillon C. (2010). Phasic vs sustained fear in rats and humans: Role of the extended amygdala in fear vs anxiety. Neuropsychopharmacology Official Publication America Collection Neuropsychopharmacology.

[bib0055] Droit-Volet S., Meck W.H. (2007). How emotions colour our perception of time. Trends in Cognitive Sciences.

[bib0060] Droit-Volet S., Mermillod M., Cocenas-Silva R., Gil S. (2010). The effect of expectancy of a threatening event on time perception in human adults. Emotion Washing DC.

[bib0065] Droit‐Volet S., Brunot S., Niedenthal P. (2004). BRIEF REPORT Perception of the duration of emotional events. Cognition & Emotion.

[bib0070] Failing M., Theeuwes J. (2016). Reward alters the perception of time. Cognition.

[bib0075] Faul F., Erdfelder E., Lang A.-G., Buchner A.G. (2007). Power 3: A flexible statistical power analysis program for the social, behavioral, and biomedical sciences. Behavior Research Methods.

[bib0080] Fayolle S., Gil S., Droit-Volet S. (2015). Fear and time: Fear speeds up the internal clock. Behavioural Processes.

[bib0085] Folta-Schoofs K., Wolf O.T., Treue S., Schoofs D. (2014). Perceptual complexity, rather than valence or arousal accounts for distracter-induced overproductions of temporal durations. Acta Psychology (Amst.).

[bib0090] Gable P.A., Poole B.D. (2012). Time flies when you’re having approach-motivated fun: Effects of motivational intensity on time perception. Psychological Science.

[bib0095] Grommet E.K. (2011). Time estimation of fear cues in human observers. Behavioural Processes.

[bib0100] Hicks R.E., Miller G.W., Gaes G., Bierman K. (1977). Concurrent processing demands and the experience of time-in-Passing. The American Journal of Psychology.

[bib0105] Horstmann G. (2015). The surprise-attention link: A review. Annals of the New York Academy of Sciences.

[bib0110] Kierkegaard S. (1957). The concept of dread.

[bib0115] Kingdom F.A.A., Prins N. (2010). Psychophysics: A practical introduction.

[bib0120] Koch G. (2008). Impaired reproduction of second but not millisecond time intervals in Parkinson’s disease. Neuropsychologia.

[bib0125] Kopec C.D., Brody C.D. (2010). Human performance on the temporal bisection task. Brain and Cognition.

[bib0130] Lake J.I., Meck W.H., LaBar K.S. (2016). Discriminative fear learners are resilient to temporal distortions during threat anticipation. Timing Time Perception Leiden Neth..

[bib0135] Lake J.I., LaBar K.S., Meck W.H. (2016). Emotional modulation of interval timing and time perception. Neuroscience and Biobehavioral Reviews.

[bib0140] Macar F., Grondin S., Casini L. (1994). Controlled attention sharing influences time estimation. Memory & Cognition.

[bib0145] Mioni G., Stablum F., Prunetti E., Grondin S. (2016). Time perception in anxious and depressed patients: A comparison between time reproduction and time production tasks. Journal of Affective Disorders.

[bib0150] Palumbo L., Ogden R., Makin A.D.J., Bertamini M. (2014). Examining visual complexity and its influence on perceived duration. Journal of Vision.

[bib0155] Prins N., Kingdom F.A.A. (2009). Palamedes: Matlab routines for analyzing psychophysical data. http://www.palamedestoolbox.org.

[bib0160] Rey A.E. (2017). Pain dilates time perception. Scientific Reports.

[bib0165] Robinson O.J., Vytal K., Cornwell B.R., Grillon C. (2013). The impact of anxiety upon cognition: Perspectives from human threat of shock studies. Frontiers in Human Neuroscience.

[bib0170] Schmitz A., Grillon C. (2012). Assessing fear and anxiety in humans using the threat of predictable and unpredictable aversive events (the NPU-threat test). Nature Protocols.

[bib0175] Simen P., Matell M. (2016). Why does time seem to fly when we’re having fun?. Science.

[bib0180] Spielberger C.D. (1983). Manual for the state-trait anxiety inventory STAI (Form Y) (‘Self-Evaluation questionnaire’).

[bib0185] Stetson C., Fiesta M.P., Eagleman D.M. (2007). Does Time Really Slow Down during a Frightening Event?. PloS One.

[bib0190] Terhune D.B., Sullivan J.G., Simola J.M. (2016). Time dilates after spontaneous blinking. Curr. Biol. CB.

[bib0195] Thomas E.A.C., Weaver W.B. (1975). Cognitive processing and time perception. Perception & Psychophysics.

[bib0200] Tipples J. (2008). Negative emotionality influences the effects of emotion on time perception. Emotion Washington DC.

[bib0205] Tipples J. (2011). When time stands still: Fear-specific modulation of temporal bias due to threat. Emotion.

[bib0210] Todd A.R., Forstmann M., Burgmer P., Brooks A.W., Galinsky A.D. (2015). Anxious and egocentric: how specific emotions influence perspective taking. Journal of Experimental Psychology General.

[bib0215] Tottenham N. (2009). The NimStim set of facial expressions: Judgments from untrained research participants. Psychiatry Research.

[bib0220] Tovote P., Fadok J.P., Lüthi A. (2015). Neuronal circuits for fear and anxiety. Nature Reviews Neuroscience.

[bib0225] Tse P.U., Intriligator J., Rivest J., Cavanagh P. (2004). Attention and the subjective expansion of time. Perception & Psychophysics.

[bib0230] van Wassenhove V., Wittmann M., Craig, Bud A.D., Paulus M.P. (2011). Psychological and neural mechanisms of subjective time dilation. Frontiers in Neuroscience.

[bib0235] Vicario C.M., Felmingham K.L. (2018). Slower time estimation in post-traumatic stress disorder. Scientific Reports.

[bib0240] Wearden J., Ferrara A. (1996). Stimulus range effects in temporal bisection by humans. The Quarterly Journal of Experimental Psychology B, Comparative and Physiological Psychology.

[bib0245] Wearden J.H., O’Rourke S.C., Matchwick C., Min Z., Maeers S. (2010). Task switching and subjective duration. The Quarterly Journal of Experimental Psychology.

[bib0250] Wiener M., Turkeltaub P., Coslett H.B. (2010). The image of time: A voxel-wise meta-analysis. NeuroImage.

